# MC-Net: a method for the construction of phylogenetic networks based on the Monte-Carlo method

**DOI:** 10.1186/1471-2148-10-254

**Published:** 2010-08-20

**Authors:** Changiz Eslahchi, Mahnaz Habibi, Reza Hassanzadeh, Ehsan Mottaghi

**Affiliations:** 1 Faculty of Mathematics, Shahid Beheshti University, G.C., Tehran, Iran; 2 School of Computer Science, Institute for Studies in Theoretical Physics and Mathematics (IPM), Tehran, Iran

## Abstract

**Background:**

A phylogenetic network is a generalization of phylogenetic trees that allows the representation of conflicting signals or alternative evolutionary histories in a single diagram. There are several methods for constructing these networks. Some of these methods are based on distances among taxa. In practice, the methods which are based on distance perform faster in comparison with other methods. The Neighbor-Net (N-Net) is a distance-based method. The N-Net produces a circular ordering from a distance matrix, then constructs a collection of weighted splits using circular ordering. The SplitsTree which is a program using these weighted splits makes a phylogenetic network. In general, finding an optimal circular ordering is an NP-hard problem. The N-Net is a heuristic algorithm to find the optimal circular ordering which is based on neighbor-joining algorithm.

**Results:**

In this paper, we present a heuristic algorithm to find an optimal circular ordering based on the Monte-Carlo method, called MC-Net algorithm. In order to show that MC-Net performs better than N-Net, we apply both algorithms on different data sets. Then we draw phylogenetic networks corresponding to outputs of these algorithms using SplitsTree and compare the results.

**Conclusions:**

We find that the circular ordering produced by the MC-Net is closer to optimal circular ordering than the N-Net. Furthermore, the networks corresponding to outputs of MC-Net made by SplitsTree are simpler than N-Net.

## Background

Phylogenetics is concerned with the construction and analysis of phylogenetic trees or networks to understand the evolution of species, populations, and individuals. Evolutionary processes such as hybridization between species, lateral transfer of genes, recombination within a population, and convergent evolution can all lead to evolutionary histories that are distinctly non-treelike. Moreover, even when the underlying evolution is treelike, the presence of conflicting or ambiguous signals can make a single tree representation inappropriate. In these situations, phylogenetic network methods can be particularly useful.

Phylogenetic network is a generalization of phylogenetic trees that can represent several trees simultaneously. For any network construction method, the conflicting signals should be represented in the network but it is vital that the network does not depict more conflict than is found in the data. At the same time, when the data fits well to a tree, the method should return a network that is close to a tree. Recently, in addition to biology, the phylogenetic networks methods are widely used for classifying different types of data such as those finding in linguistics, music, etc. There are many different methods to construct phylogenetic trees or networks which are based on distance matrix such as ME (minimum evolution) [1], LS (least squares) [2,3], NJ (neighbor-joining) [4], AddTree [5], N-Net (neighbor-net) [6] and Q-Net [7]. All these methods are called distance-based methods.

ME is one of the most well-known methods. It was first introduced by Kidd and Sgamarella-Zonta [1]. Given a distance matrix, the ME principle consists of selecting the tree whose length (sum of its branch lengths) is minimal among all tree topologies for taxa. Comparative studies of tree-building methods show that ME generally is an accurate criterion for selecting a true tree. Nei and Rzhetsky have shown that ME principle is statistically consistent when branch lengths are assigned by ordinary least-squares (OLS) fitting [8]. In the OLS framework, we simply minimize

$$\sum_{i,j\in X}{\left(d_{ij}\;-\delta_{i,j}\right)}^{2},$$

where *δ_ij _*is an estimation of input *d_ij _*and X is the set of taxa. In fact, the main goal is to find a tree whose induced metric is closer to *d_ij_*. The LS was first introduced in [2] and [3].

Nearly 20 years have passed by since the landmark paper in Molecular Biology and Evolution introducing NJ [4]. The method has become the most widely used method for building phylogenetic trees from distances. Steel and Gascuel showed that NJ is a greedy algorithm for ME principle [9]. The N-Net is a hybrid of NJ and split decomposition [10]. It is applicable to data sets containing hundreds of taxa. The N-Net is an algorithm for constructing phylogenetic networks.

Split decomposition, implemented in SplitsTree [11], decomposes the distance matrix into simple components based on weighted splits. These splits are then represented using a special type of phylogenetic network called split network. The N-Net works in a similar way: it first produces a circular ordering from distance matrix and then constructs a collection of weighted splits. Dan Levy and Lior Patcher showed that the N-Net is a greedy algorithm for the traveling salesman problem that minimizes the balanced length of the split system at every step and it is optimal for circular distance matrices [12]. Balanced minimum evolution (BME) is designed under the ME principle [13]. The BME is a special version of the ME principle where tree length is estimated by the weighted least squares [13].

In this work, we introduce MC-Net algorithm (Monte-Carlo Network algorithm) which works in a similar way: First, it finds a circular ordering for taxa, based on Monte-Carlo with simulated annealing, it then extracts splits from the circular ordering and uses non-negative least squares for weighting splits. We compare the results of the N-Net and the MC-Net for several data sets.

### Preliminaries

**Figure 1:**
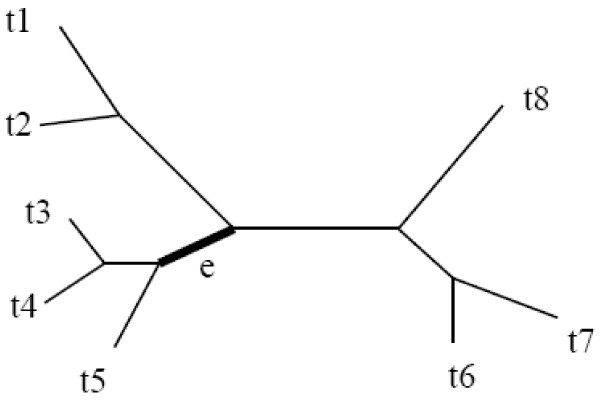
**The split *S *= *A*|*B *is obtained by removing the edge *e *of *T***.

A *split *of a given set *X *of taxa is a bipartition of the set *X *into two non-empty subsets of *X*. A split is called *trivial *if one of the two subsets contains only one taxon. Let T be a non-empty tree. Let the leaves of the *T *are labeled by the set of taxa, *X *={*x*_1_,...,*x_n_*}. Every edge *e *of *T *defines a split *S = A|B*, where *A *and *B *are two sets of taxa contained in the two components of *T - e*. For example, Figure 1 shows an eight-leaf tree. Removing the edge e from the tree produces two sets of leaves

$$A=\{t_{3},t_{4},t_{5}\}\;and\;B=\{t_{1},t_{2},t_{6},t_{7},t_{8}\}.$$

In an edge-weighted tree, the weight of each edge is assigned to its corresponding split. The *Phyletic distance *between any two taxa *x *and *y *in an edge-weighted tree is the sum of the weights of the edges along the path from *x *to *y*. Hence, the phyletic distance between *x *and *y *equals the sum of split weights for all those splits in which *x *and *y *belong to separate components.

Two different splits *S*_1 _= *A*_1_|*B*_1_, and *S*_2 _= *A*_2_|*B*_2_, are *compatible*, if one of the following conditions holds:

$$A_{1}\subseteq A_{2},A_{1}\subseteq B_{2},B_{1}\subseteq A_{2}\;or\;B_{1}\subseteq B_{2}.$$

A collection of splits is called compatible, if all possible pairing of splits are compatible. A compatible collection of splits is represented by a phylogenetic tree [14,15]. Dress and Huson introduced SplitsTree to display more complex evolutionary patterns [16]. For a set of incompatible splits, SplitsTree outputs the split network using bands of parallel edges.

*Circular collection of splits *is a mathematical generalization of compatible collections of splits. Formally, a collection of splits of *X *is circular if there exists an ordering *x*_1_,⋯,*x_n _*of *X *such that every split is of the form {*x_i_, x*_*i*+1_,⋯,*x_j_*}|*X *- {*x_i_,⋯,x_j_*} for some *i *and *j*, 1 ≤ *i *≤ *j *≤ *n*. A Compatible collection of splits are always circular [10]. On the other hand, the class of circular collection of splits contains the class of the collection of splits corresponding to a tree. Andreas Dress and Daniel Huson proved that circular collections of splits always have a planar splits graph representation [16]. A distance matrix is circular (also called Kalmanson) if it is the phyletic distances for a circular collection of splits with positive weights. Because compatible splits are circular, treelike distances are circular too [6].

As mentioned above, the ME principle consists of selecting a tree whose length is minimal. In fact, the ME principle is equivalent to finding a circular ordering *σ *= {*x*_*σ*(1)_,...,*x*_*σ*(*n*)_} in order to find the minimum of the function *η*

(1) $$\begin{array}{c} \eta :\sum \to ℜ\\ \eta (\sigma )=d(x_{\sigma (1)},x_{\sigma (n)})+\sum_{k=1}^{n-1}d(x_{\sigma (k)},x_{\sigma (k+1)}) \end{array}$$

Where Σ is the set of all circular orderings of taxa *x*_1_,...,*x_n_*. We call function *η *the *energy function*, and any circular ordering that minimizes *η *is called the *optimal circular ordering*.

## Methods

There are a number of different methods for constructing various kinds of phylogenetic networks. A phylogenetic network can be constructed from a collection of weighted splits. N-Net uses circular ordering to construct a collection of weighted splits. Since finding an optimal circular ordering is an NP-hard problem, so we introduce a heuristic algorithm based on the Monte-Carlo method to find optimal circular ordering. The MC-Net seeks to find an optimal circular ordering from the distance matrix and then extracts a collection of weighted splits based on that ordering.

### Algorithms

In this section, a new algorithm called the MC-Net, is presented to construct a set of weighted splits for taxa set *X *= {*x*_1_,...,*x_n_*}with a given distance matrix. The MC-Net consists of two steps. In the first step, we find a circular ordering. In the second step, the splits which are obtained from the circular ordering are weighted. The core of the first step contains two procedures, namely, INITIAL and the Monte-Carlo. The INITIAL is a greedy algorithm to obtain a circular ordering, namely, the initial circular ordering. The INITIAL works in the following way:

Suppose *x*_*σ*(1)_,...,*x*_*σ*(*k*) _are ordered and let $\bar{x}$ be an element of *S *= *X *- {*x*_σ(1)_,...,*x*_σ(*k*)_} such that

$$d\left(\bar{x},r\right)=\min\{d\left(x,r\right)|x\in S,r\in \{x_{\sigma (1)},x_{\sigma (k)}\}\}.$$

If *r *= *x*_σ(1)_, we consider the new ordering $\bar{x},x_{\sigma (1),\ldots ,}x_{\sigma (k)}$. Otherwise the ordering $x_{\sigma (1),\ldots ,}x_{\sigma (k)},\bar{x}$ is considered. This process stops when all taxa are ordered.

The second procedure, or the Monte-Carlo procedure, relies on random iteration to find the optimal circular ordering. The Monte-Carlo algorithm starts its movement from the initial circular ordering, *σ_0 _*For each circular ordering *σ*, we define the neighborhood of *σ*, *N *(*σ*), by:

$$\begin{array}{c} N(\sigma )=\{\tilde{\sigma }\in \sum |\exists k;\;2\leq k\leq n-1;\\ \;\tilde{\sigma }=\{x_{\sigma (1),\ldots ,}x_{\sigma (k-1),}x_{\sigma (k+1),\ldots ,}x_{\sigma (n)},x_{\sigma (k)}\}\}, \end{array}$$

where Σ is the set of all circular orderings.

We choose *σ*_1 _∈ *N *(*σ_0_*) randomly. if *η *(*σ*_1_) ≤ *η *(*σ_0_*), then the system moves into ordering *σ*_1_. However we allow non-greedy movements for the system in order to avoid having the system trapped in local minima. In other words, if *η *(*σ*_1_) >*η *(*σ_0_*), then the system moves into ordering σ_1 _with a small probability $e\frac{-\eta (\sigma_{1})+\eta (\sigma_{0})}{T}$, where *T *is a constant temperature. For each temperature, these movements are carried out *t *times. To compute the minimum energy we allow this process to continue until the temperature drops to zero (see the appendix for more details). Pseudo code of the Monte-Carlo algorithm is shown in Table 1. It is notticeable that the second procedure can start from any circular ordering other than the one obtained by the INITIAL procedure.

**Table 1:** Pseudo code of the Monte-Carlo algorithm with simulated annealing

**Input: *T *initial temperature**
***σ_0 _*initial ordering**
***T_low _*low temperature**
***t *constant number**

*σ =σ_0_*
**While ***T > T_low_*
**Repeat ***t *time
choose random $\tilde{\sigma }\in N\left(\sigma \right)$
**If **$\eta \left(\tilde{\sigma }\right)\leq \eta \left(\sigma \right)$
$\sigma =\tilde{\sigma }$
**Else**
*x *= *random*(0, 1)
**If **$x<e\frac{-\eta (\tilde{\sigma })+(\sigma )}{T}$
$\sigma =\tilde{\sigma }$
*T *= T * 0.9
**Return ***σ *and *η*(*σ*)

In the final step, we use the least squares algorithm to weight the splits of obtained circular ordering. Let *A *be the matrix with rows indexed by pairs of taxa and columns indexed by splits. Then for each pair of texa *i *and *j *and for each split *k*, *A_ij,k _*is defined by:

$$A_{ij,k}=\{\begin{array}{ll} 1 & \text{if }i\text{ and }j\text{ are on opposite of split }k;\\ 0 & \text{otherwise}. \end{array}$$

The matrix *A *= [*A_ij,k_*] is full rank [17].

Let *d *= (*d*_12_, *d*_13_,...,*d*_(*n*-1)*n*_) be an *n*(*n - *1)*/2 *dimensional vector corresponding to rows of *A *where *d_ij _*is obtained by distance matrix. Let *b *be the weight vector of splits, then the phyletic distance vector is *p = Ab*.

Now, the ordinary least squares(OLS) is used to estimate *b *by the following standard formula

(2) $$b={\left(A^{\prime }A\right)}^{-1}A^{\prime }d.$$

If we discard splits with negative weights and leave the remaining splits unchanged, the weight of the remaining splits are often grossly overestimated. Similar to the N-Net algorithm, we compute the optimal least square estimates with a non-negative constraint. In this paper, we use the FNNLS algorithm [18].

## Results and Discussion

In this section, we compare the results of the MC-Net and the N-Net on some data sets. We use SplitsTree4 program [19] for drawing phylogenetic networks. Due to the limitation of space, we insert only six figures in this article.

### Data sets

One of the data sets, a collection of 110 Salmonella MLST Data, was obtained from authors of the N-Net. The other data sets presented as the examples in SplitsTree4 program (version 4.10): Its(46 taxa), Jsa (46 taxa), Mammals (30 taxa), Primates (12 taxa), Rubber (23 taxa), Dolphins (36 taxa) and Myosin (143 taxa).

### Optimal threshold for cooling coefficient and *T*_*low*_

**Figure 2:**
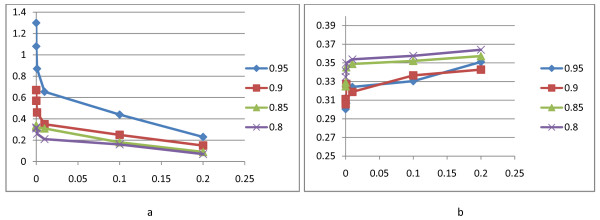
**The value of energy function (b) and running time of algorithm (a) for each *T_low _*for JSA data**.

There are two parameters, *T_low _*and cooling coefficient, in the Monte-Carlo procedure. We first adjust *T_low _*between 10^5 ^and 0.2 to obtain the best cooling coefficient. The value of energy function and running time of algorithm for each *T_low _*for JSA data are given in Figure 2 (for the other data sets, the figures are the same as JSA). According to Figure 2, when cooling coefficient is 0.95, running time of the algorithm compared to other coefficients increases considerably. On the other hand, the value of energy function for 0.95 or 0.9 as a cooling coefficient is significantly better than the other cooling coefficients. Hence, we conclude that the best value of energy function with respect to running time of the algorithm is achieved when cooling coefficient is 0.9 and *T*_*low *_< 10^-3^.

## Results

The initial test for performance of our method is done by calculating the value of energy function for circular orderings obtained by the MC-Net and the N-Net (Table 2). The first two rows of Table 2 show that in all data sets except Salmonella, the value of energy function for the MC-Net is less than those obtained from the N-Net. The interesting feature of the MC-Net algorithm is in finding different circular orderings by changing initial ordering. So, the MC-Net algorithm could take the circular ordering obtained by the N-Net as initial ordering. The third row of Table 2 shows the values of energy function for circular orderings achieved by the MC-Net with the circular ordering obtained by the N-Net as an initial ordering. For four data sets, Its, Rubber, Salmonella, Myosin, the third row indicates better results than the first row. But for the other data sets, the conclusions mentioned above are the vice versa.

**Table 2:** Values of energy function: the values of energy function for circular orderings obtained by the N-Net, the MC-Net and the MC-Net with initial ordering of the N-Net.

Data set	Its	Jsa	Mammals	Primates
**N-Net**	0.4096	0.2808	4.4275	2.1465
**MC-Net**	0.4079	0.2728	4.4172	2.1410
**start N-Net**	0.3979	0.2767	4.4202	2.1410

				

Data set	Rubber	Dolphins	Salmonella	Myosin

**N-Net**	0.7723	2.2	0.2546	43.8199
**MC-Net**	0.7596	2.1667	0.2575	43.8019
**start N-Net**	0.7547	2.2	0.2515	43.6935

Another test for the performance of our method is comparing the number of splits obtained by both the algorithms. In Table 3, the number of splits of circular orderings obtained by the MC-Net and the N-Net on different data sets are shown. In all data sets the number of splits obtained by the MC-Net is less than the N-Net except Primates. In this case, these two numbers are equal.

**Table 3:** The number of splits obtained by the MC-Net and the N-Net for all data sets.

Data set	Its	Jsa	Mammals	Primates
**N-Net**	110	83	103	34
**MC-Net**	105	78	99	34

				

Data set	Rubber	Dolphins	Salmonella	Myosin

**N-Net**	55	67	107	520
**MC-Net**	53	62	90	507

Let *d *be the input distance vector and *P *and *P' *are the phyletic distance vector of weighted splits obtained by the MC-Net and the N-Net, respectively. In Table 4, the value of norm of *P - d *and *P' - d *for each data set are shown. The norm of *P - d *is less than *P' - d *in all data sets even in Primates. It means that the results of the MC-Net algorithm give better approximation for input distance vector.

**Table 4:** The value of norm for all data sets.

Data set	Its	Jsa	Mammals	Primates
**N-Net**	0.0444	0.0329	0.0717	0.0385
**MC-Net**	0.0358	0.0292	0.0648	0.0358

				

Data set	Rubber	Dolphins	Salmonella	Myosin

**N-Net**	0.0362	0.1068	0.0487	0.0291
**MC-Net**	0.0316	0.1019	0.0405	0.0207

**Figure 3:**
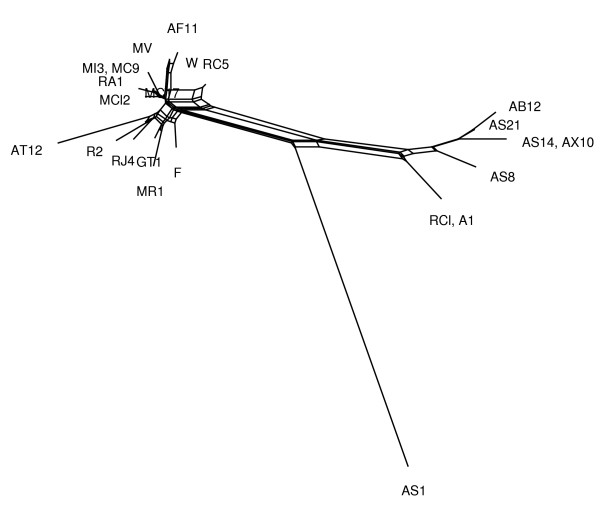
**The N-Net network for the Rubber data set**.

**Figure 4:**
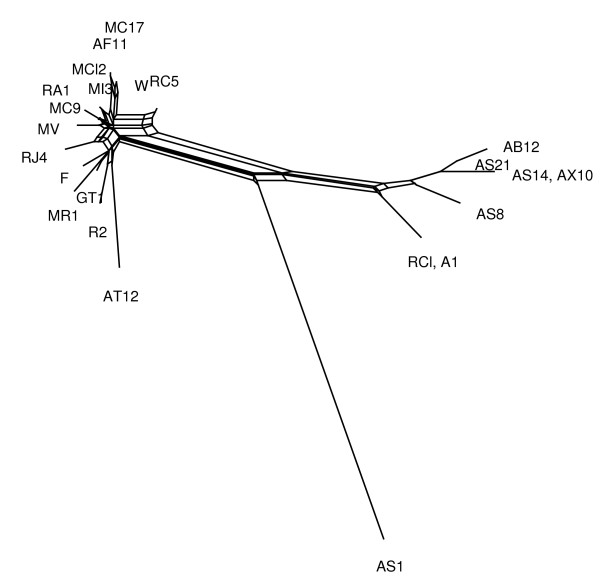
**The MC-Net network for the Rubber data set**.

**Figure 5:**
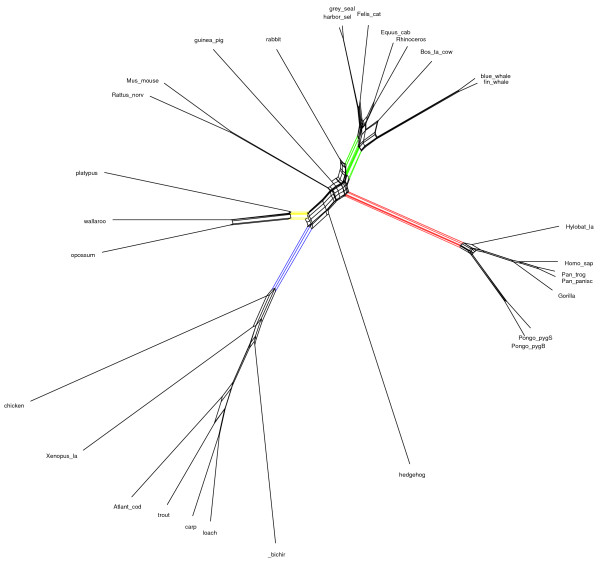
**The N-Net network for the Mammal data set**.

**Figure 6:**
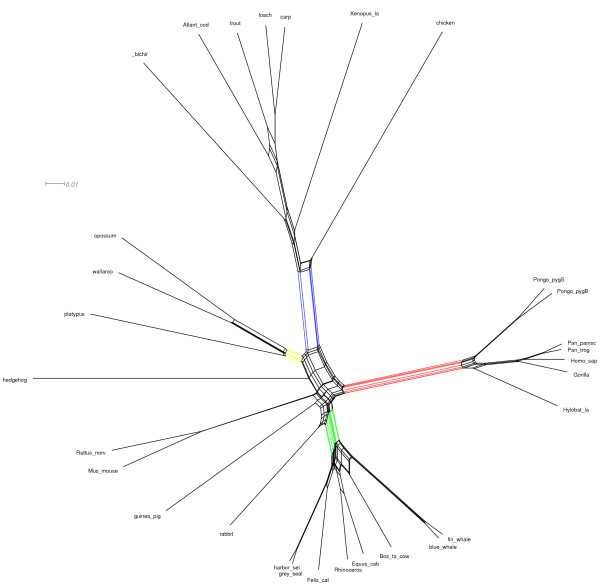
**The MC-Net network for the Mammal data set**.

**Figure 7:**
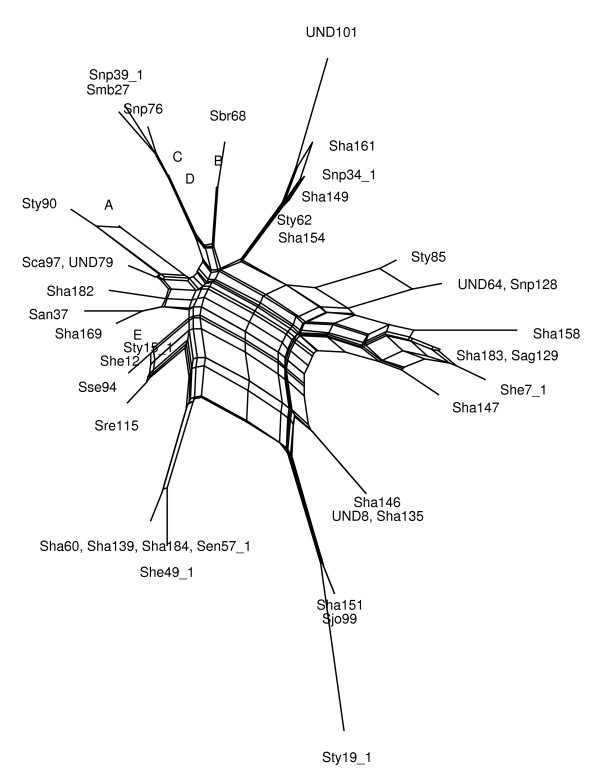
**The N-Net network for the Salmonella data set**. Group A includes the isolates Sty54, Sty54*, Sty2, She9, Sty87, Snp40*, Sty13, Snp41*, Sen5, Sha160, Sha141, Sty20*, Sha58, Sse18, Sha71, Sty31. Group B includes the isolates Sty61, Sha148, Smb-17, Sag75, Sha124. Group C includes the isolates UND3, Sha150, Sha173, Sen23*, Sha153, Sha140, San96, Sen30*, Sen24*, Sha138, Sha176, Sha130, Sha164, Sha157, Sen29*, Sca93, Sha122, Sht20, Sha186. Group D includes the isolates She3, Sha50, Sse95, Sha56, Sen24, Sen34, Sha177, Sty13*, Swo44, Sty86, Ste41, Sha77, UND80. Group E includes the isolates Ssc40, Sse28, Sty89, Sty15*, Ske69, UND110, Sha49, Sen4, Sha48, Sha165, Sty92, Snp33*, Sty52, UND109, Sha131, Sha102, Sty6, Sha175.

**Figure 8:**
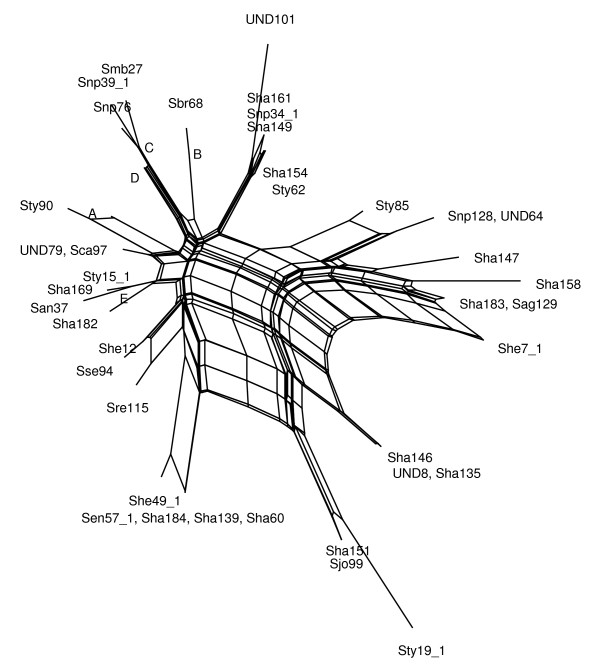
**The MC-Net network for the Salmonella data set**. Group A includes the isolates Sty54, Sty54*, Sty2, She9, Sty87, Snp40*, Sty13, Snp41*, Sen5, Sha160, Sha141, Sty20*, Sha58, Sse18, Sha71, Sty31. Group B includes the isolates Sty61, Sha148, Smb-17, Sag75, Sha124. Group C includes the isolates UND3, Sha150, Sha173, Sen23*, Sha153, Sha140, San96, Sen30*, Sen24*, Sha138, Sha176, Sha130, Sha164, Sha157, Sen29*, Sca93, Sha122, Sht20, Sha186. Group D includes the isolates She3, Sha50, Sse95, Sha56, Sen24, Sen34, Sha177, Sty13*, Swo44, Sty86, Ste41, Sha77, UND80. Group E includes the isolates Ssc40, Sse28, Sty89, Sty15*, Ske69, UND110, Sha49, Sen4, Sha48, Sha165, Sty92, Snp33*, Sty52, UND109, Sha131, Sha102, Sty6, Sha175.

To illustrate difference between two algorithms, we present some examples of networks obtained by both the MC-Net and the N-Net using SplitsTree4 (Figures 3,4,5,6, 7 and 8). It is obvious that both algorithms give the same classification of taxa and exhibit the same major splits. For example, in Figures 5 and 6, we highlight some edges such that by removing the same-colored edges, the same clustering of taxa is obtained. But according to what we see in Tables 3 and 4, split networks obtained by the MC-Net are less complicated than split networks obtained by the N-Net. It means that the networks obtained by the MC-Net have less noise than the networks obtained by the N-Net. According to Corollary 1 (see Appendix), when t approaches to 1, the MC-Net finds optimal circular ordering with the probability 1. We examined our algorithm on several treelike distance matrices and it returned corresponding trees quickly. The MC-Net has been implemented in Matlab and is available for download at http://bioinf.cs.ipm.ac.ir/softwares/mc.net.

## Conclusions

In this work, we propose an algorithm, MC-Net, which is a distance based method for constructing phylogenetic networks. The MC-Net scales well and can quickly produce detailed and informative networks for large number of taxa. We compare the performance of the MC-Net with the N-Net on eight different data sets. We have shown (Tables 2, 3 and 4) that the MC-Net performs better than the N-Net for almost test cases and the networks obtained by the MC-Net are simpler than the N-Net with the same major splits. The N-Net is a part of SplitsTree program. So, the results of the MC-Net could be used in SplitsTree program too.

## Authors' contributions

CE, RH and EM performed initial studies. MH designed the algorithm. RH and EM analysis the data sets. All authors participated in the writing of the manuscript. All authors read and approved the final manuscript.

## Appendix

Let *S *= {*E*_1_,...,*E_s_*} be a finite set of states, and consider a physical process having these discrete states at time *t*. A Markov chain is a stochastic model of this system, such that the state of system at time *t *+ 1 depends only on the state of system at time *t*.

Consider *X*_0_, *X*_1_,..., be a collection of Markov random variables, such that *X_n _*is the state of the system at time *n*. Let *p_ij _*be the probability that the system enters into the state *E_j _*from the state *E_i_*, where *i, j *∈ {1,...,*s*} The matrix *P *= (*p*_*ij*_)_1≤*i*, *j*≤*s *_is called *transition matrix*. A probability distribution *q *= (*q*_1_,...,*q_s_*) such that *q_i _*is the probability that system starts its movement from the state *E_i _*, is called *initial probability distribution*. A *Markov chain *is a stochastic model *X*_0_, *X*_1_,..., such that *X_t _*is the state of the system at time *t*. For each *i *and *j *in {1,2,...,*s*};

$$\begin{array}{c} prob\left(X_{0}=E_{i}\right)=q_{i},\\ prob\left(X_{t+1}=E_{j}|X_{t}=E_{i}\right)=p_{ij}. \end{array}$$

The Markov chain is *irreducible*, if for all *i, j *∈ {1,...,*s*} there exists *n *> 0 such that $p_{ij}^{\left(n\right)}>0$, where

$$\forall \alpha \;p_{ij}^{\left(n\right)}=prob(X_{n+\alpha }=E_{j}|X_{\alpha }=E_{i}).$$

In other words, the Markov chain is irreducible, if there exist *n *such that the probability that the system enters into the state *E_j _*from the state *E_i _*after *n *times is positive. The irreducible Markov chain is called aperiodic, if for some *n *≥ 0 and some state *E_j_*,

$$\begin{array}{c} prob\left(X_{n}=E_{j}|X_{0}=E_{j}\right)>0\\ \&\\ prob\left(X_{n+1}=E_{j}|X_{0}=E_{j}\right)>0. \end{array}$$

### Theorem 1(Convergence to stationary Markov chain, [20])

If the Markov chain is irreducible and aperiodic then

$$\underset{t\to \infty }{\text{lim}}prob\left(X_{t}=E_{j}\right)=\pi_{j}\;\;j=1,\ldots ,s$$

such that *π *= (*π*_1_,...,*π_s_*) is a unique probability distribution and $\pi_{j}=\sum_{i=1}^{s}\pi_{i}p_{ij}$.

The probability distribution is π is called *stationary probability *of the Markov chain.

It means that if *P *is the transition matrix and *P*^(*t*) ^is the *t^th ^*power of *P*, when *t *→ ∞ the *j^th ^*column of transition matrix is approximately equal to *π_j_*. In the Monte-Carlo algorithm, a special kind of Markov chain is used. Let Σ be the finite set of states and $q=\left(\frac{1}{\left|\Sigma \right|},\ldots ,\frac{1}{\left|\Sigma \right|}\right)$ is the initial probability distribution. For each state *i *the neighborhood of *i*, *N*(*i*), is defined as the set of all the states that are reachable from *i *by one movement. In this system the set of neighborhoods have to satisfy the following properties:

1. *i*, ∉ *N*(*i*).

2. *i *∈ *N*(*j*) ⇔ *j *∈ *N*(*i*)

3. if *i ≠ j*, then there exit *i*_1_,*i*_2_,...,*i*_1 _∈ Σ such that

$$i\in N\left(i_{1}\right),i_{1}\in N\left(i_{2}\right),\ldots ,i_{l}\in N\left(j\right).$$

The matrix $P^{T}={\left(p_{ij}^{T}\right)}_{i,j\in \Sigma }$ is defined as the transition matrix by

$$p_{ij}^{T}=\{\begin{array}{ll} \frac{1}{\left|N(i)\right|} & \text{if }j\in N(i)\;and\;\eta (j)\leq \eta (i),\\ \frac{e^{-(\eta (j)-\eta (i))/T}}{\left|N(i)\right|} & \text{if }j\in N(i)\;and\;\eta (j)>\eta (i),\\ 1-\sum_{k\in \Sigma ,k\neq i}p_{ik}^{T} & \text{if }i=j,\\ 0 & \text{otherwise,} \end{array}$$

where *T *is a positive constant number (constant temperature). The third property of the neighborhood shows that this Markov chain is irreducible. Also, if $P_{ii}^{T}>0$ and *P^T ^*contains non-negative entries then ${\left(P^{T}\right)}_{ii}^{\left(t\right)}>0$ for all *t *≥ 0. So, it is a finite, aperiodic and irreducible Markov chain. The theorem 1 shows that for each constant temperature *T *and *i *∈ Σ, there exists a stationary probability distribution $\pi_{i}^{T}$ such that:

$$\underset{t\to \infty }{\lim}prob(X_{t}=i)=\pi_{i}^{T},$$

Where $\pi_{i}^{T}=\frac{e^{-\frac{\eta (i)}{T}}}{\sum_{j\in \sum }e^{-\frac{\eta (j)}{T}}}$ (see page 45 in [20]).

**Proposition 1**. Let ${\left(\pi_{i}^{T}\right)}_{i\in \Sigma }$ be a probability distribution such that:

$$\pi_{i}^{T}=\frac{e^{-\frac{\eta (i)}{T}}}{\sum_{j\in \sum }e^{-\frac{\eta (j)}{T}}}$$

and suppose that *m*_0 _= min{*η*(*i*) | *i *∈ Σ} and, *η_0 _*= {*i *∈ Σ | *η*(*i*) = *m*_0_} then for each $i\in \Sigma ,\underset{T\to 0^{+}}{\lim}\pi_{i}^{T}=\pi_{i}^{0}$, where

$$\pi_{i}^{0}=\{\begin{array}{ll} \frac{1}{\left|\eta_{0}\right|} & \text{if }i\in \eta_{0};\\ 0 & \text{otherwise}. \end{array}$$

*Proof*: The proof is presented in [20] (claim 2.8 and claim 2.9).

**Corollary 1**. Let Σ be the finite set of states, then for each *i *∈ Σ we have

$$\underset{T\to 0^{+}}{\lim}\underset{t\to \infty }{\lim}prob(X_{t}=i)=\pi_{i}^{0}.$$

The corollary 1 illustrates that by cooling temperature (*T *→ 0^+^), system enters into one of the states of *η*_0 _with the probability 1 after *t *(*t *→ ∞) time. In this article, we define the set of all circular orderings of taxa as the finite set of states. Our definition of neighborhood in the MC-Net satisfies in three properties of neighborhood and every elements of *η*_0 _is an optimal circular ordering. Therefore, the MC-Net yields a circular ordering with approximately minimal energy function.
